# C-reactive protein- and clinical symptoms-guided strategy in term neonates with early-onset sepsis reduced antibiotic use and hospital stay: a quality improvement initiative

**DOI:** 10.1186/s12887-020-02426-w

**Published:** 2020-11-20

**Authors:** Johan Gyllensvärd, Fredrik Ingemansson, Elisabet Hentz, Marie Studahl, Anders Elfvin

**Affiliations:** 1 Department of Pediatrics, Region Jönköping County, Jönköping, Sweden S- 553 05, Jönköping, Sweden; 2 Department of Clinical and Experimental Medicine, Linköping University, Linköping, Sweden; 3 Department of Pediatrics, Institute of Clinical Sciences, Sahlgrenska Academy, University of Gothenburg, Gothenburg, Sweden; 4 Department of Pediatrics, Sahlgrenska University Hospital, Gothenburg, Sweden; 5 Department of Infectious Diseases, Institute of Biomedicine, Sahlgrenska Academy, University of Gothenburg, Gothenburg, Sweden; 6 Region Västra Götaland, Department of Infectious Diseases, Sahlgrenska University Hospital, Gothenburg, Sweden

**Keywords:** Bacterial infection, C-reactive protein, Neonatal sepsis, Antibiotic therapy, Antibiotic stewardship, Quality improvement

## Abstract

**Background:**

Early-onset sepsis (EOS) is a potentially life-threatening complication of birth. Clinical symptoms are often unspecific and biomarkers have low predictive values for EOS. Therefore, clinical suspicion often leads to antibiotic therapy in neonates with a negative blood culture. In the study we evaluated if a quality improvement initiative could reduce unwarranted antibiotic use in a safe way in term neonates with culture-negative sepsis.

**Methods:**

The quality improvement initiative included new treatment guidelines and were introduced on 11 June 2018. The guidelines included C-reactive protein- and clinical symptoms-guided decision-making and shorter intravenous antibiotic therapy. All term neonates treated for EOS at Ryhov Hospital, Jönköping, Sweden were studied before (period 1: 2016–2017) and after the introduction of the new guidelines (period 2: 11 June 2018 to 30 Sept 2019).

Laboratory and clinical data were analysed.

**Results:**

There were 7618 term neonates in period 1 and 5005 term neonates in period 2. We identified 140 (1.8%) EOS in period 1 and 97 (1.9%) EOS in period 2. During period 1 and 2, there were 61 (61/140, 44%) and 59 (59/97, 61%) EOS neonates, respectively, who met the criteria for shorter antibiotic treatment. The number of positive blood cultures were seven (0.92/1000 live births) and five (1.0/1000 live births) in period 1 and 2. The median C-reactive protein were 52 mg/L (37–62) in period 1 and 42 mg/L (31–56) in period 2 in the group who met the criteria of the guidelines. The duration of antibiotic therapy (Median: seven vs. five days, *p* < 0.001) and hospital stay (Median: seven vs. five days, *p* < 0.001) as well as healthcare costs (decreased by €122,000/year) was reduced in the group who met the criteria after the introduction of the guidelines.

**Conclusion:**

C-reactive protein- and clinical symptoms-guided decision-making for EOS significantly decreased the duration of antibiotic therapy and hospital stay, and hence reduced healthcare costs, with no reinfection in a cohort of term infants.

**Trial registration:**

Trial registration number: ISRCTN29535824. Date of registration: 28 May 2020. Retrospectively registered.

**Supplementary Information:**

The online version contains supplementary material available at 10.1186/s12887-020-02426-w.

## Key notes

- This study compared antibiotic therapy and hospital stay in early-onset sepsis before (period 1) and after (period 2) the introduction of new guidelines including CRP- and clinical symptoms-guided decision-making.
- The study included 61 neonates in period 1 and 59 neonates in period 2 who met the criteria of the guidelines.
- The duration of antibiotic therapy and hospital stay as well as healthcare costs decreased between the periods without any reinfection.

## Background

Early-onset sepsis (EOS) is a potentially life-threatening complication of birth [1]. Delayed antimicrobial therapy of sepsis increases the risk of morbidity and mortality, making it important to recognise and diagnose sepsis early [2]. The most common risk factors associated with EOS are maternal group B streptococcal (GBS) bacteriuria or GBS colonisation in the current pregnancy, a previous neonate with invasive GBS disease, maternal fever and prolonged rupture of membranes (≥18 h) [2–5]. Clinical symptoms are often unspecific and biomarkers have low predictive values for EOS [6]. Therefore, clinical suspicion often leads to antibiotic therapy in uninfected neonates. The incidence of culture-confirmed EOS among infants born at term is approximately 0.4–0.8 in 1000 live births in most high-income countries [7–13]. The number of neonates receiving antibiotic therapy for culture-negative sepsis is six to 16 times higher than neonates receiving therapy for culture-confirmed sepsis [13].

Consequently, neonates with culture-negative sepsis are associated with high antibiotic use in neonatal units [7, 14]. Overuse of antibiotics may lead to increased colonisation with antibiotic resistant bacteria [15]. Antibiotic therapy in term neonates has also been associated with increased risks of asthma, wheezing, food allergy and childhood obesity [16–19].

Hence, it is important to minimise unnecessary antibiotic treatment. One way to decrease the antibiotic exposure is to discontinue antibiotic therapy in the absence of a culture-confirmed infection.

C-reactive protein (CRP) is one of the most widely used and studied acute-phase reactants for neonatal sepsis as it is fast, cost-effective and simple [20]. Using CRP as a guide to help clinicians to discontinue antibiotic therapy might be useful. To our knowledge there is little evidence to determine guidelines concerning the duration of antimicrobial therapy in infants with culture-negative sepsis and elevated CRP [3, 6, 13].

In order to discontinue antibiotic therapy earlier in term neonates and reduce unwarranted antibiotic use the neonatal intensive care unit (NICU) in Jönköping implemented a quality improvement (QI) project including a modified version of the clinical guidelines from the NICU of Queen Silvia’s Children’s Hospital in Gothenburg, Sweden. This local guideline is now in clinical use in Jönköping. National guidelines are under development but not yet implemented.

The aim of this study was to evaluate whether the introduction of the new treatment guidelines in the NICU resulted in a decreased number of days with antibiotic treatment without increasing the rate of reinfection or readmission.

## Methods

### Study design and setting

This QI project compared antibiotic use of term neonates treated for EOS before and after the introduction of new treatment guidelines (introduced 11 June 2018) at one single centre level 2 NICU in Jönköping, Sweden. The new guidelines were developed in order to help physicians´ decision to discontinue antibiotics in term infants when they met the criteria of the guidelines. In the study we decided a priori to evaluate the guidelines by comparing data from 24 months before and 15–16 months after the introduction of the guidelines. Period 1 was between 1 January 2016 and 31 December 2017 and period 2 was between 11 June 2018 and 30 September 2019. Infants born during period 1 were considered a baseline group. The period from 1 Jan 2018 to 10 June 2018 was considered an educational period where local physicians modified the clinical guidelines from the NICU of Queen Silvia’s Children’s Hospital in Gothenburg, Sweden. This was made within the QI framework. The physicians and nursing staff where educated about the new treatment guidelines and therefore the implementation went smoothly.

The primary outcome was number of days with antibiotic treatment. The secondary outcome was reinfection or readmission to hospital within three days after completed antibiotic treatment. Other outcomes were hospital stay and healthcare costs. Healthcare costs were calculated based on the cost per day of care [21].

There is no screening for maternal GBS bacteriuria or rectovaginal colonisation with GBS in asymptomatic pregnant women. Only women with clinical symptoms are evaluated with a urine culture and if they have GBS bacteriuria they are treated with antibiotics. Intrapartum prophylactic antibiotics is routinely given to mothers if there is prolonged rupture of membranes (> 18 h), GBS bacteriuria, a previous GBS infected child and maternal fever. There is no specific guideline at the unit when to start antibiotic therapy in term infants with symptoms and/or risk factors for EOS, but both clinical symptoms and a significant increase of CRP or Interleukin-6 (IL-6) are needed. Antibiotic therapy is never started only based on risk factors alone. In the study there were no specific time for CRP measurements after onset of therapy, it was up to the attending physician to decide when it was appropriate.

At the neonatal unit in Jönköping antibiotic treatment is routinely discontinued after 36–72 h if symptoms and laboratory findings are not in accordance with EOS. Those infants where not diagnosed with any infection, and therefore not included in the present study. If the blood culture was negative after 48–72 h, antibiotic treatment continued only if the physician had strong clinical reasons to believe it was an EOS.

The unit provides a regional service for preterm neonates with a GA of ≥27 weeks. The unit aimed to obtain a minimum blood volume of 1 mL for blood cultures.

### Definitions

In the study, EOS was defined as onset of symptoms within 72 h of life. Culture-positive EOS in neonates was defined as growth of a recognised pathogen in blood cultures in combination with clinical signs of infection. Culture-negative EOS in neonates was defined, in the study, as infants who were diagnosed with an infection and had clinical signs of infection (including respiratory instability, temperature instability, cardiovascular instability, gastrointestinal symptoms, irritability and lethargy), elevated biomarkers for infection with CRP > 20 mg/L and, or, IL-6 > 350 ng/L and subsequent antibiotic therapy. No sepsis was defined as cases who received antibiotics but were never diagnosed with an infection. The attending physician decided whether it was an infection or not.

### Patient material and procedures

All neonates who were admitted to the neonatal unit at the Ryhov County Hospital during period 1 and 2 with any antibiotic treatment during the first three days of life, and the diagnostic codes for neonatal sepsis P36 (P36.0–36.9) or infection specific for the perinatal period P39 (P39.8–39.9), according to the International Classification of Diseases, 10th revision (ICD-10-SE) were included in the study. The neonates were identified by using the Region Jönköping County electronic medical record searching tool (Diver) and the Swedish Neonatal Quality Register (SNQ).

Before the new guidelines were introduced, neonates with both culture-positive and culture-negative EOS used to be treated with at least seven days of intravenous antibiotics at the NICU. In neonates where intravenous access was not accessible infants received oral suspension antibiotics instead.

According to the new treatment guidelines the intravenous antibiotic therapy can be withdrawn after three days followed by administration of oral suspension antibiotic (Amoxicillin, 20 mg/kg three times a day) for two more days in term neonates meeting the criteria of the new treatment guidelines.

The new guidelines contained the following criteria;

- term neonate (week 37 + 0 to week 41 + 6), and
- no need of intensive care (including invasive respiratory support or cardiovascular support) initially when the antibiotic treatment started, and
- the neonate appeared well on day 3, and
- the blood culture was not positive on day 3, and
- maximum CRP-value of 80 mg/L decreasing by at least 50% during the first three days.

For extra safety, the infants included in the study also received a routine visit on day 7 for clinical assessment and control of CRP.

In the study infants with EOS received penicillin G 50 mg/kg every 8–12 h and amikacin 15 mg/kg every 24 h.

### Data extraction

The following details were reviewed from the medical record: when the antibiotic treatment was initiated and terminated, drugs prescribed, duration of antibiotic treatment and hospital stay, any readmission or reinfection within three days after ending antibiotic treatment, clinical symptoms at initiation of antibiotic treatment, whether the neonate appeared well on day 3, 30-day mortality and relevant laboratory data (CRP, IL-6 and blood culture results).

Background data were collected on: sex, birthweight, gestational age (GA), Apgar scores, time of rupture of the membranes and arterial pH at birth. A positive blood culture was categorised either as a proven infection or as a contamination. If a positive blood culture result was noted in the patient record as a contaminant it was defined as a contaminant. Growth of two or more species in a blood culture was considered as contamination.

Inclusion criteria: Term neonates in gestational week 37 + 0 to week 41 + 6 and clinically diagnosed as EOS that had initiated antibiotic treatment within 72 h of life. Exclusion criteria: Term neonates who received prophylactic antibiotic treatment and not because of an EOS.

In the study population we were primarily interested in the group who met the criteria of the new treatment guidelines referred to as the new guideline group.

Length of antibiotic treatment as well as hospital stay were counted in days. When the neonate received only one dose of intravenous antibiotic or one dose of oral suspension antibiotic during a day, it was counted as one day with antibiotic treatment. On occasions when the neonate received both intravenous and oral suspension antibiotic treatment during the same day it was counted as one day with intravenous antibiotic treatment. Regardless of when the neonate was admitted or discharged from the NICU, it was counted as a full day of care.

### Ethics and statistical analysis

The regional ethical committee of Linköping approved this study, dnr 2018/503–31. Data were analysed using SPSS version 25 (IBM, Armonk NY, USA) and Statistica version 13.3 (TIBCO Software Inc., Palo Alto CA, USA). The study was registered at isrctn.com, number ISRCTN29535824.

Comparison between groups was calculated with the Mann-Whitney U-test and Chi-square test. Data are shown using medians and interquartile ranges (IQR) and also means and standard deviations (SD). Categorical data are shown as percentages. This study was designed to obtain a power of 90% and a significance level of 5%, in order to detect a reduction from seven to five days of antibiotic therapy. To obtain this power, significance level and desired effect size, a sample of 60 patients in each treatment group were required.

## Results

**Fig. 1:**
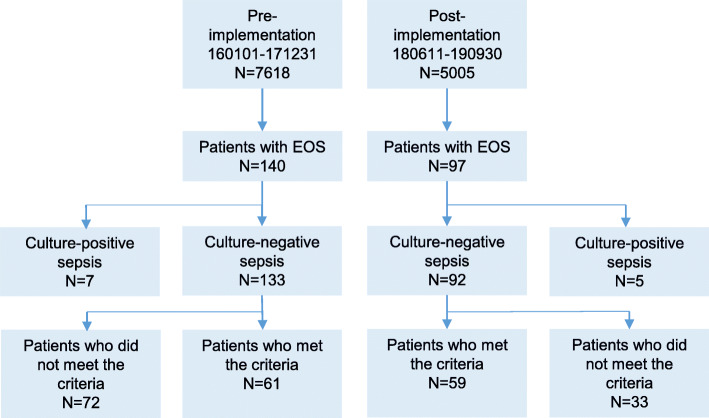
Study flow chart. Number of term infants born and treated for early-onset sepsis (EOS) in the region during the study period. Pre- and post-implementation were before and after the introduction of the new antibiotic treatment guidelines

In the Region Jönköping County 7618 term infants (week 37 + 0 to week 41 + 6) were born during period 1 and 5005 term infants during period 2 (Fig. 1). There were 62 and 41 infants with unknown GA in period 1 and period 2, respectively. Patients characteristics on neonates admitted to the NICU and diagnosed with EOS and who met the criteria of the guidelines are presented in Table 1.

**Table 1:** Patients characteristics on infants with early-onset sepsis who met the criteria of the new guidelines

	Pre-implementation Period 1 (***n*** = 61)	Post-implementation Period 2 (***n*** = 59)	***P***-value
Sex			
Female	26/61 (43%)	27/59 (46%)	0.73
Gestational age, weeks	40 (39–40)	40 (39–41)	0.91
Gestational days	283 (279–286)	281 (278–287)	0.99
Birthweight, (grams), mean (SD)	3768 (559)	3899 (497)	0.26
Arterial cord pH	7.20 (7.10–7.24) (*n* = 54)	7.22 (7.13–7.26) (*n* = 50)	0.080
Apgar score			
5 min < 7	6/61 (9.8%)	3/59 (5.1%)	0.32
10 min < 7	4/61 (6.6%)	2/59 (3.4%)	0.43
Rupture of membranes			
0–12 h	43/58 (74%)	37/53 (70%)	0.34
> 12–24 h	10/58 (17%)	7/53 (13%)	0.48
> 24 h-1 week	5/58 (8.6%)	9/53 (17%)	0.23
CRP mg/L	52 (37–62)	42 (31–56)	0.10
IL-6 ng/L	444 (152–1441) (*n* = 53)	322 (127–1470) (*n* = 54)	0.61

Term infants treated for early-onset sepsis (EOS) pre- and post-implementation of new antibiotic treatment guidelines. Period 1 and 2 include term infants with culture-negative sepsis who met the criteria of the guidelines. Data are median (IQR) or *n*/total (%) except for birthweight which is mean (SD). Numbers are less than n in each group where data were not available. *CRP* C-reactive protein, *IL-6* Interleukin-6

During period 1 we identified 185 term neonates who were admitted to the NICU in the first three days of life and treated with antibiotic therapy (Table 2). There were 45 neonates who were excluded because they were not classified as having sepsis (*n* = 43) or they only received prophylactic antibiotics (*n* = 2). During period 2 we identified 128 term neonates who were admitted to the NICU in the first three days of life and treated with antibiotic therapy (Table 2). One neonate was excluded because he was transferred to another hospital at one day of age as a result of heart failure and 30 neonates were excluded because they were not having sepsis. The study included 140 and 97 neonates during period 1 and 2, respectively, with culture-positive EOS or culture-negative EOS (Fig. 1, Additional files 1 and 2).

**Table 2:** Data on all infants who received antibiotics during the study periods

	Pre-implementation Period 1 (***n*** = 7618)	Post-implementation Period 2 (***n*** = 5005)	***p***-value
Any antibiotic treatment	185 (2.4)	128 (2.6)	0.72
Diagnosis, *n*(%)			
No sepsis	45 (24)	31 (24)	0.98
Culture-negative sepsis who met the criteria	61 (33)	59 (46)	0.019
Culture-negative sepsis who did not meet the criteria	72 (39)	33 (26)	0.016
Culture-positive sepsis	7 (3.8)	5 (3.9)	0.96

All term neonates (week 37 + 0 to week 41 + 6) who received antibiotics or were classified as culture-negative sepsis or culture-positive sepsis at the unit during the study period. Patients with culture-negative sepsis were divided into two groups. One group who met the criteria of the new guidelines and another group who did not

There were 61 infants in period 1 who met the criteria of the guidelines (not yet implemented guidelines) (Table 1); 79/140 neonates did not meet the criteria, of whom 16 met the criteria except that CRP was not measured on day 3. There were 59 infants in period 2 who met the criteria of the guidelines (Table 1); 38/97 neonates did not meet the criteria.

**Fig. 2:**
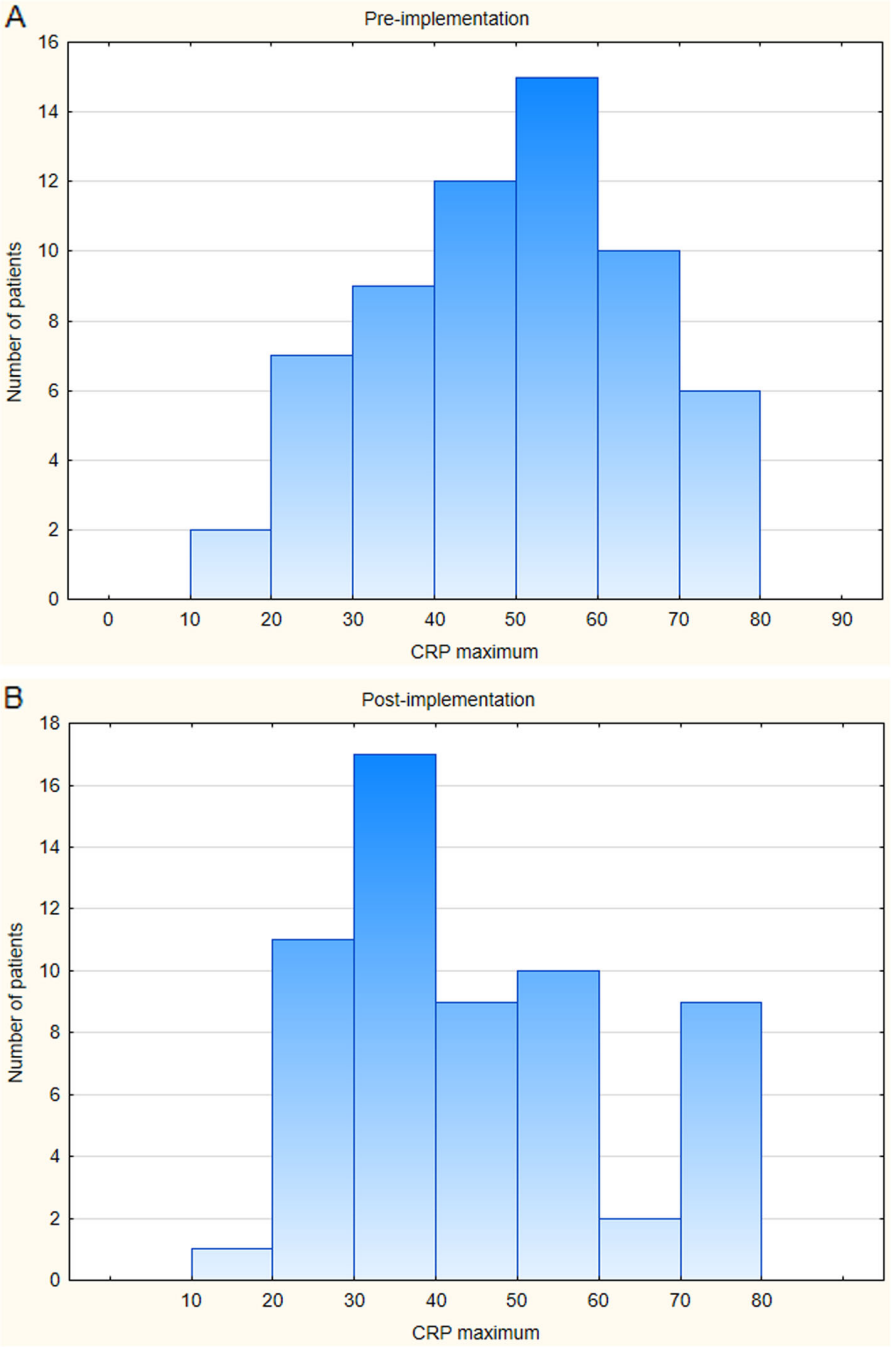
**a** Number of patients pre-implementation who met the criteria of the new guidelines and distribution of their maximum CRP-level in mg/L. **b** Number of patients post-implementation who met the criteria of the new guidelines and distribution of their maximum CRP-level in mg/L.

In the study there were seven blood cultures in period 1 and five blood cultures in period 2 that were positive (Tables 3 and 4). The distribution of CRP levels in patients who met the criteria for shorter antibiotic treatment are shown in Fig. 2a and b, respectively.

**Table 3:** Bacterial distribution of blood culture-confirmed early-onset sepsis pre-implementation

	Number	Percentage	Incidence rate
Group B Streptococcus	1	14	0.13
Streptococcus other	1	14	0.13
Stafylococcus species	3	43	0.39
Other Gram-negative	1	14	0.13
Enterococcus species	1	14	0.13
Escherichia coli	0	0	0
Total	7	100	0.92

Incidence rate among term infants per 1000 per year. Calculated by dividing cases with population size (7618) and multiplying by 1000

**Table 4:** Bacterial distribution of blood culture-confirmed early-onset sepsis post-implementation

	Number	Percentage	Incidence rate
Group B Streptococcus	2	40	0.40
Streptococcus other	2	40	0.40
Stafylococcus species	0	0	0
Other Gram-negative	0	0	0
Enterococcus species	0	0	0
Escherichia coli	1	20	0.20
Total	5	100	1.0

Incidence rate among term infants per 1000 per year. Calculated by dividing cases with population size (5005) and multiplying by 1000

During period 2, there was one neonate who met the criteria of the guidelines but had a GBS-positive nasopharyngeal culture and was therefore clinically assessed as having a culture positive infection. The child received seven days of antibiotic treatment and was not included in the new guideline group. During period 1 and 2, there were two and one patients, respectively, who met the criteria of the guidelines but had growth of two species in their blood cultures. They were treated as having a culture positive sepsis by the attending physician. All of these patients had antibiotic therapy for seven to ten days and were not included in the new guideline group. In period 1 and 2, there were three (3/133, 1.6%) and one (1/92, 0.78%) patients, respectively, who got shorter antibiotic therapy than five days due to clinical improvements.

### Primary endpoint

Among the patients who met the criteria for the new guidelines there was a significant difference between period 1 and 2 in the number of days of antibiotic therapy (Table 5). The median (IQR) number of days with antibiotic therapy in period 1 was seven (7-8) and five (5-6) in period 2 (Table 5). There was also a significant difference between period 1 and 2 in the number of days of intravenous antibiotic therapy (Table 5). The median (IQR) number of days with intravenous antibiotic therapy among the patients who met the criteria in period 1 was five (5-7) and three (3-4) in period 2 (Table 5).

**Table 5:** Antibiotic therapy and length of hospital stay in patients with early-onset sepsis

	Pre-implementation Period 1 (***n*** = 61)	Post-implementation Period 2 (***n*** = 59)	***P***-value
AB-days	7 (7–8)	5 (5–6)	*P* < 0.001
IV-days	5 (5–7)	3 (3–4)	*P* < 0.001
Oral-days	0 (0–4)	2 (2–3)	*P* = 0.064
H-days	7 (6–8)	5 (4–6)	*P* < 0.001

Term infants treated for early-onset sepsis (EOS) pre- and post-implementation of new antibiotic treatment guidelines. Period 1 and 2 include term infants with culture-negative sepsis who met the criteria of the guidelines. Data are median (IQR). *AB-days* days with antibiotic treatment, *IV-days* days with intravenous antibiotic treatment, *Oral-days* days with oral solution antibiotic treatment, *H-days* Hospital stay in days

### Secondary endpoints

There was no reinfection within three days after discontinuation of antibiotics in period 1 or 2 among patients who met the criteria for shorter antibiotic therapy. There was only one reinfection in period 1 in one neonate who did not meet the criteria. There was one readmission in Period 2. This neonate had no clinical symptoms but had a rising CRP (8-17 mg/L) at the routine visit on day 6. During observation at hospital for one night no antibiotics were given and CRP decreased to 10 mg/L the following day. It was the only one of all neonates who had an elevated CRP at the return visit. There was one infant who died during period 2 at the age of 10 h because of an intrauterine infection, multi-organ failure and severe asphyxia in the group that did not meet the criteria; otherwise there were no deaths in any group within 30 days of birth.

In the new guideline group there was a significant difference between period 1 and period 2 in the median number of days of the hospital stay (Table 5). The median (IQR) duration of hospital stay in period 1 was seven days (6-8) and five days (4-6) in period 2 (Table 5).

Consequently there were in median two days saved per patient, and at a cost of €1350 per day this corresponds to €2700 per patient. During period 2, a total of 59 patients met the criteria, resulting in a total saving of €159,000, which corresponds to an annual saving of about €122,000.

## Discussion

This study showed that new treatment guidelines including CRP- and clinical symptoms-guided decision-making significantly reduced the duration of antibiotic therapy and hospital stay in term infants with culture-negative EOS. There was no reinfection or study-related mortality in the groups who met the criteria. Combining CRP measurements, negative blood culture, the neonate’s symptoms and clinical signs supports antimicrobial stewardship and helps physicians to decide whether to discontinue antibiotic treatment earlier during the disease course in neonates who meet the criteria.

Proven neonatal infection is quite unusual, but antibiotic treatment for culture-negative EOS is more common [13]. One study in California, USA, showed a 40-fold variation in patient-days of antibiotic use with similar rates of proven infections in a comparison of 127 NICUs, which indicates that physicians beliefs or the unit’s policy influence the start and duration of antibiotic treatment [22].

One of the reasons could be that there is no consensus definition for culture-negative neonatal sepsis [13]. This leads to differences in antibiotic treatment between different neonatal units and physicians. Previous studies have used different definitions where some of them use, as one of the criteria, antibiotic treatment for more than three to five days [13]. In the study there were three and one patients in period 1 and 2, respectively, who received less than five days of therapy and it could not be excluded that they were misclassified as culture-negative EOS.

A number of studies have previously been carried out using different methods, all trying to reduce the antibiotic treatment in a safe way in neonates receiving antibiotic therapy because of culture-negative EOS. We found only two studies using CRP-guided decision-making to reduce the duration of antibiotic therapy in neonates. One study had a cut-off value for CRP of less than 10 mg/L [23] and the other one required a normalisation of the CRP [24] before discontinuing antibiotic therapy, which makes it difficult to compare them to our study.

Stocker and colleagues conducted a multicentre, randomised trial and used procalcitonin-guided decision-making to reduce antibiotic therapy in neonates with suspected EOS, and showed a significant reduction in the duration of antibiotic therapy (55 h vs. 65 h) and length of stay between the procalcitonin-guided therapy group and the standard therapy group, with no proven reinfection or study-related deaths in either group [25]. In their study 42% of the infants were classified as category 4 (710/1710, 42%, infection unlikely) out of totally four risk categories, according to their algorithm for assessment of risk classification. However, a comparison with our study is not possible since our NICU do not initiate antibiotic treatment at all in neonates with similar risk assessment as in category 4. In addition, neonates in category 2 (infection probably) were treated with antibiotics for 7 to 21 days despite having a negative blood culture. Several neonates in our study, who in their study would have been classified as category 2 and thus treated for 7–21 days with antibiotics, met our criteria for shorter antibiotic therapy.

Stocker and collegues found 27 proven bacterial infections in 1710 neonates treated with antibiotics (Number needed to treat (NNT) = 63) [25]. A nationwide study from Norway found 91 proven bacterial infection in 3964 term neonates treated with antibiotics (NNT = 44) [7]. In comparison, we found 12 proven bacterial infections in 313 term neonates treated with antibiotics (NNT = 26) in our study indicating a more restrictive initiation of antibiotic treatment. Cantey and coworkers used an autostop at 48 h for empiric antibiotics and reduced the antibiotic days of therapy by 27% with no difference in safety outcomes, but neonates had a significantly longer median length of stay [14]. Dretvik and colleagues compared the duration of antibiotic therapy in suspected EOS in three Norwegian NICUs before and after implementation of an evidence-based guideline which was developed in order to reduce unwarranted antibiotic treatment. They did not find a significant reduction in antibiotic therapy duration in neonates treated for suspected EOS but the overall antibiotic use was reduced without affecting safety [26].

If continuing with antibiotic therapy for longer than 36 h despite negative blood cultures, there is a recommendation, according to the National Institute of Health and Care Excellence (NICE) guidelines, to review the infant at least every 24 h. Also the level of the initial clinical suspicion of infection, the infant’s clinical progress and current condition should be reconsidered each day, including assessment of “reassuring” levels and trends of CRP when deciding timepoint for withdrawing of antibiotic treatment [6]. Unfortunately the guidelines lack a discussion of “reassuring” CRP values in this context. One report showed longer duration of antibiotic treatment, more investigations and longer hospital stay after introducing the new NICE guidelines [27].

Consequently, there are different approaches to safely reducing antibiotic therapy in neonates with culture-negative sepsis, although not all of them lead to reduced antibiotic therapy. Our study showed one way to reduce antibiotic therapy, length of hospital stays, and the cost of care.

The incidence of blood culture positive EOS (0.92/1000 live births in period 1 and 1.0/1000 live births in period 2) was higher in our study compared to other studies from high-income countries, although the incidence of GBS was 0.13/1000 live births in period 1 and 0.40/1000 live births in period 2 which is similar to or lower than the reported in other studies [10, 13, 28]. An obvious explanation to the differences in the incidence rate in our study is hard to find.

The QI-project was implemented smoothly in our NICU without any extra staffing or cost. One explanation could be that we are a relatively small NICU with physicians who are used to follow new guidelines. However, we acknowledge that this could be a challenge in other units.

There are several limitations to our study. The QI study design has inherent limitations. The power calculation was calculated on an expected reduction in the duration of antibiotic treatment and therefore does not rule out rare side effects, which would have required a much larger population.

The arbitrary chosen level of 80 mg/L in CRP as a cut-off, was used in the present study and is still in use in the local clinical guideline. The upcoming Swedish national guidelines have suggested 60 mg/L in CRP as a cut-off as an extra precaution. We did not see any negative consequences of including patients up to 80 mg/L in CRP in our study. We also took into account the dynamics of CRP development, symptoms at disease onset, the blood culture result and the clinical condition of the infant when deciding to discontinue intravenous antibiotic treatment. Additionally, CRP values up to at least 40–50 mg/L in non-infected neonates have been seen [29], which raises the question whether some of the infants in the study were not infected at all. Small volumes of blood obtained, low levels of bacteremia and maternal antibiotic therapy before or during birth may be an explanation for culture-negative EOS [13]. IL-6 as a biomarker for infection in EOS is not as well-known as CRP and the cut-off value for infection is uncertain. In this study a cut-off at 350 ng/L for IL-6 was used, which in general is a low threshold for infection. Among the patients who met the criteria of the new guidelines there was no death within 30 days of birth in period 1 or 2. Period 2 represents another year, so the resistance pattern could have affected the result, although we did not see any changes in the resistance pattern in the control cultures that had been taken every week at the NICU for several years. The single centre setting limits the possibility to extrapolate to other NICUs. We only included term neonates, so the results cannot be extrapolated to preterm neonates.

The strength of the study is that we included all neonates attending the NICU and receiving antibiotic therapy for EOS during the study period.

In our study, the infants received oral solution antibiotic treatment after the initial intravenous antibiotic therapy as an extra precaution. However, the effect of oral solution antibiotic treatment is uncertain. In future studies we intend to evaluate the effect of exclusively intravenous antibiotic therapy for 48–72 h in a similar group of patients as in this study.

## Conclusions

CRP- and clinical symptoms-guided decision-making for EOS significantly decreased the duration of antibiotic therapy and hospital stay, and hence reduced healthcare costs, with no reinfection in a cohort of term neonates in Sweden.

## Supplementary Information

**Additional file 1.** Patients characteristics on all infants with early-onset sepsis. Term infants treated for early-onset sepsis (EOS) pre- and post-implementation of new antibiotic treatment guidelines. Period 1 and 2 include term infants with both culture positive and culture-negative sepsis. Data are median (IQR) or n/total (%) except for birthweight which is mean (SD). Numbers are less than n in each group where data were not available. CRP=C-reactive protein; IL-6 = Interleukin-6.

**Additional file 2.** Antibiotic therapy and length of hospital stay in patients with early-onset sepsis. Term infants treated for early-onset sepsis (EOS) pre- and post-implementation of new antibiotic treatment guidelines. Period 1 and 2 include term infants with both culture positive and culture-negative sepsis. Data are median (IQR). AB-days = days with antibiotic treatment; IV-days = days with intravenous antibiotic treatment; Oral-days = days with oral solution antibiotic treatment; H-days = Hospital stay in days.

## Data Availability

The datasets generated and/or analysed during the current study are not publicly available due to that the ethics committee specifically state that no data, which can identify a patient can be publicly available but are available from the corresponding author on reasonable request.

## Abbreviations

CRP: C-reactive protein
EOS: Early-onset sepsis
GA: Gestational age
GBS: group B streptococcal
ICD-10: International Classification of Diseases 10th revision
IL-6: Interleukin-6
IQR: Interquartile range
NICU: Neonatal intensive care unit
NICE: National Institute of Health and Care Excellence
NNT: Number needed to treat
QI: Quality improvement
SD: Standard Deviation
SNQ: Swedish Neonatal Quality Register
