# Supplementary material for: C-reactive protein- and clinical symptoms-guided strategy in term neonates with early-onset sepsis reduced antibiotic use and hospital stay: a quality improvement initiative
Source: BMC Pediatr. 2020 Nov 20;20:531. doi: 10.1186/s12887-020-02426-w (PMC7678045; doi:10.1186/s12887-020-02426-w)
Supplement: Supplementary file 1 — Additional file 1. Patients characteristics on all infants with early-onset sepsis. Term infants treated for early-onset sepsis (EOS) pre- and post-implementation of new antibiotic treatment guidelines. Period 1 and 2 include term infants with both culture positive and culture-negative sepsis. Data are median (IQR) or n/total (%) except for birthweight which is mean (SD). Numbers are less than n in each group where data were not available. CRP=C-reactive protein; IL-6 = Interleukin-6. [file 12887_2020_2426_MOESM1_ESM.docx]

**Additional file 1** Patients characteristics on all infants with early-onset sepsis

|  | **Pre-implementation**  **Period 1**  **(n=140)** | **Post-implementation**  **Period 2**  **(n=97)** | **p-value** |  |
| --- | --- | --- | --- | --- |
| Sex  Female | 49/140 (35%) | 39/97 (40%) | 0.42 |  |
| Gestational age, weeks | 40 (39-41) | 40 (39-41) | 0.68 |  |
| Gestational days | 283  (278-287) | 282  (278-288) | 0.80 |  |
| Birthweight, (grams), mean (SD) | 3796  (550) | 3863  (512) | 0.46 |  |
| Arterial cord pH | 7.18  (7.09-7.24)  (n=124) | 7.20  (7.11-7.25)  (n=82) | 0.24 |  |
| Apgar score  5min<7  10min<7 | 22/139 (16%)  13/139 (9.4%) | 14/97 (14%)  10/97 (10%) | 0.68  0.71 |  |
| Rupture of membranes  0-12 h  >12-24 h  >24 h-1 week | 89/133 (67%)  26/133 (20%)  18/133 (14%) | 58/88 (66%)  18/88 (20%)  12/88 (14%) | 0.56  1.0  0.91 |  |
| CRP mg/L | 59  (42-85) | 53  (38-75) | 0.13 |  |
| IL-6 ng/L | 597  (160-2331)  (n=130) | 527  (135-1699)  (n=88) | 0.39 |  |
| Culture positive infection | 7/132 (5.3%) | 5/94 (5.3%) | 1.0 |  |
| Term infants treated for early-onset sepsis (EOS) pre- and post-implementation of new antibiotic treatment guidelines. Period 1 and 2 include term infants with both culture positive and culture-negative sepsis. Data are median (IQR) or n/total (%) except for birthweight which is mean (SD). Numbers are less than n in each group where data were not available. CRP=C-reactive protein; IL-6 =Interleukin-6. | | | | |
