# Supplementary material for: C-reactive protein- and clinical symptoms-guided strategy in term neonates with early-onset sepsis reduced antibiotic use and hospital stay: a quality improvement initiative
Source: BMC Pediatr. 2020 Nov 20;20:531. doi: 10.1186/s12887-020-02426-w (PMC7678045; doi:10.1186/s12887-020-02426-w)
Supplement: Supplementary file 2 — Additional file 2. Antibiotic therapy and length of hospital stay in patients with early-onset sepsis. Term infants treated for early-onset sepsis (EOS) pre- and post-implementation of new antibiotic treatment guidelines. Period 1 and 2 include term infants with both culture positive and culture-negative sepsis. Data are median (IQR). AB-days = days with antibiotic treatment; IV-days = days with intravenous antibiotic treatment; Oral-days = days with oral solution antibiotic treatment; H-days = Hospital stay in days. [file 12887_2020_2426_MOESM2_ESM.docx]

**Additional file 2** Antibiotic therapy and length of hospital stay in all patients with early-onset sepsis

|  | **Pre-implementation**  **Period 1**  **(n=140)** | **Post-implementation**  **Period 2**  **(n=97)** | **p-value** |  |
| --- | --- | --- | --- | --- |
| AB-days | 8 (7-9.5) | 6 (5-7) | P<0.001 |  |
| IV-days | 6 (5-7) | 4 (3-6) | P<0.001 |  |
| Oral-days | 0 (0-3) | 2 (1-3) | P=0.15 |  |
| H-days | 8 (6-9) | 6 (5-8) | P<0.001 |  |
| Term infants treated for early-onset sepsis (EOS) pre- and post-implementation of new antibiotic treatment guidelines. Period 1 and 2 include term infants with both culture positive and culture-negative sepsis. Data are median (IQR). AB-days= days with antibiotic treatment; IV-days= days with intravenous antibiotic treatment; Oral-days= days with oral solution antibiotic treatment; H-days= Hospital stay in days. | | | | |
